# Engineering and adaptive laboratory evolution of *Escherichia coli* for improving methanol utilization based on a hybrid methanol assimilation pathway

**DOI:** 10.3389/fbioe.2022.1089639

**Published:** 2023-01-10

**Authors:** Qing Sun, Dehua Liu, Zhen Chen

**Affiliations:** ^1^ Key Laboratory of Industrial Biocatalysis (Ministry of Education), Department of Chemical Engineering, Tsinghua University, Beijing, China; ^2^ Tsinghua Innovation Center in Dongguan, Dongguan, China; ^3^ Center for Synthetic and Systems Biology, Tsinghua University, Beijing, China

**Keywords:** methanol, *Escherichia coli*, synthetic methylotrophy, xylulose monophosphate pathway, adaptive laboratory evolution

## Abstract

Engineering *Escherichia coli* for efficient methanol assimilation is important for developing methanol as an emerging next-generation feedstock for industrial biotechnology. While recent attempts to engineer *E. coli* as a synthetic methylotroph have achieved great success, most of these works are based on the engineering of the prokaryotic ribulose monophosphate (RuMP) pathway. In this study, we introduced a hybrid methanol assimilation pathway which consists of prokaryotic methanol dehydrogenase (Mdh) and eukaryotic xylulose monophosphate (XuMP) pathway enzyme dihydroxyacetone synthase (Das) into *E. coli* and reprogrammed *E. coli* metabolism to improve methanol assimilation by combining rational design and adaptive laboratory evolution. By deletion and down-regulation of key genes in the TCA cycle and glycolysis to increase the flux toward the cyclic XuMP pathway, methanol consumption and the assimilation of methanol to biomass were significantly improved. Further improvements in methanol utilization and cell growth were achieved *via* adaptive laboratory evolution and a final evolved strain can grow on methanol with only 0.1 g/L yeast extract as co-substrate. ^13^C-methanol labeling assay demonstrated significantly higher labeling in intracellular metabolites in glycolysis, TCA cycle, pentose phosphate pathway, and amino acids. Transcriptomics analysis showed that the expression of *fba*, *dhak,* and part of pentose phosphate pathway genes were highly up-regulated, suggesting that the rational engineering strategies and adaptive evolution are effective for activating the cyclic XuMP pathway. This study demonstrated the feasibility and provided new strategies to construct synthetic methylotrophy of *E. coli* based on the hybrid methanol assimilation pathway with Mdh and Das.

## 1 Introduction

Methanol is a promising non-food feedstock for the fermentation industry due to its abundance, low price, and a high degree of reduction (Wang et al., 2020b). Methanol can be produced from the greenhouse gases methane and CO_2_, thus conversion of methanol to value-added chemicals *via* green biological processes may provide an attractive approach toward carbon neutrality (Zhu et al., 2020). Although natural methylotrophs such as *Methylobacterium extorquens* (Yuan et al., 2021) and *Bacillus methanolicus* (Brito et al., 2021) have been engineered to produce several chemicals, the relatively slow cell growth, incomprehensive understanding of cellular metabolism and the lack of effective genetic engineering tools significantly hinder the systematic engineering of these organisms toward real industrial applications (Zhu et al., 2020). Alternatively, engineering of well-characterized fast-growing microbial chassis such as *Escherichia coli* as synthetic methylotrophs by introducing heterologous methanol assimilation pathways has attracted broad attention in recent years (Gregory et al., 2022; Keller et al., 2022).

Natural methylotrophs oxidize methanol to formaldehyde by different types of methanol dehydrogenase (Mdh) or alcohol oxidase (AOX), which are further assimilated *via* three main pathways, namely the ribulose monophosphate (RuMP) pathway, the serine cycle, and the xylulose monophosphate (XuMP) pathway (Zhang et al., 2017). Since the RuMP pathway is considered to be the most energy-efficient pathway (Supplementary Table S1) (Gregory et al., 2022), large efforts have been made to engineer *E. coli* (Chen et al., 2018; Keller et al., 2022) or *Corynebacterium glutamicum* (Wang et al., 2020a) to assimilate methanol by introducing heterologous NAD-dependent Mdh and the RuMP pathway enzymes 3-hexulose-6-phosphate synthase (Hps) and 6-phospho-3-hexuloisomerase (Phi). Despite great successes in heterologous expression and optimization of the RuMP pathway, it is still very challenging to convert sugar heterotrophs to efficient methylotrophs due to the poor kinetics of heterologous enzymes, metabolic imbalance of synthetic RuMP cycle (e.g., insufficient ribulose 5-phosphate generation), and toxicity of formaldehyde resulting in DNA-protein crosslinking (Chen et al., 2020). Most synthetic methylotrophs require the supplement of other sugars (e.g., glucose (Bennett et al., 2020b), gluconate (Meyer et al., 2018), xylose (Chen et al., 2018)), pyruvate (Yu and Liao, 2018) or nutrients [amino acids (Gonzalez et al., 2018)] as a co-substrate to support cell growth. Recently, Liao’s group and Vorholt’s group have achieved great success in engineering *E. coli* for autonomous methylotrophy *via* the RuMP pathway by combining rational design and adaptive laboratory evolution (ALE) respectively (Chen et al., 2020; Keller et al., 2022). Although both engineered strains showed a growth rate comparable with natural methylotrophs (a doubling time of ∼8 h), the obtained growth rate and methanol consumption rate are still not satisfied for industrial application. Artificial one-carbon assimilation pathways, such as the reductive glycine pathway (rGlyP) (Kim et al., 2020) and the synthetic homoserine pathway (He et al., 2020), have also been proposed and experimentally verified. However, these proposed pathways still serve as proof of concept and large efforts are required to realize efficient autonomous methylotrophy. Thus, the design and engineering of efficient fast-growing synthetic methylotrophs *via* different pathways are still highly desirable for practical application.

The XuMP pathway from methylotrophic yeast is another highly efficient formaldehyde assimilation pathway (Zhu et al., 2020) while heterologous expression of the XuMP pathway in prokaryotic microorganisms has not been widely explored. The native methylotrophic yeasts such as *Pichia pastoris* can efficiently utilize methanol to obtain very high optical density. Although the energy efficiency of the natural XuMP pathway with O_2_-dependent alcohol oxidase is lower than the RuMP pathway (Whitaker et al., 2015), it is possible to increase the energy efficiency by combining eukaryotic dihydroxyacetone synthase (Das) with prokaryotic NAD-dependent Mdh. The hybrid methanol assimilation pathway can generate key glycolytic intermediate glyceraldehyde 3-phosphate (GAP) with only two enzymatic steps and the energy efficiency of the pathway is equal to the RuMP pathway when fructose-6-phosphate aldolase (Fsa) and transaldolase (Tal) are used for xylulose 5-phosphate (Xu5P) generation (Supplementary Table S1). Recently, De Simone et al. demonstrated the first example of heterologous expression of Mdh and Das in *E. coli* and showed that methanol can be integrated into several intermediates in central metabolism when cells were cultured with methanol and xylose as co-substrates (De Simone et al., 2020). However, the engineered strain showed low incorporation of ^13^C-methanol into the pentose phosphate pool and proteinogenic amino acids, indicating that the regeneration of Xu5P should be further improved to increase the efficiency of the cyclic XuMP pathway.

In this study, we attempted to increase the efficiency of the hybrid methanol assimilation pathway in *E. coli* by combining rational design and ALE. We showed that methanol consumption and the biomass yield on methanol can be increased by enforcing the metabolic flux toward the XuMP cycle. Especially, we showed that the final evolved strain can utilize methanol in the presence of a low concentration (0.1 g/L) of yeast extract and ^13^C-methanol can be incorporated into glycolytic, pentose phosphate, and tricarboxylic acid cycle (TCA) intermediates, as well as free intracellular amino acids. Genome and transcriptome sequencing were also employed to clarify the potential reasons for improved methanol assimilation. This study demonstrated that it is possible to develop a methylotroph of *E. coli via* the hybrid methanol assimilation pathway with heterologous Mdh and Das.

## 2 Materials and methods

### 2.1 Bacterial strains and plasmids

All strains and plasmids used in this study are listed in Table 1. *E. coli* DH5α was used for routine cloning. The starting *E. coli* SIJ488 was derived from *E. coli* MG1655 carrying genome-integrated gene deletion machinery (Jensen et al., 2015). High-copy expression vector pTrc99a was used for the construction of the hybrid methanol assimilation pathway.

**TABLE 1:** Strains and plasmids used in this study.

Strain or plasmid	Description	Sources
Plasmids		
pTrc99a	High-copy plasmid, ColE1 ori, Amp^r^	Lab stock
pTrc99a-mdh-das	pTrc99a with *mdh* gene from *Acinetobacter garner* and *das* gene from *Pichia angusta*	This study
pTrc99a-mdh-das-antigapA	pTrc99a with *mdh* gene from *Acinetobacter garner*, *das* gene from *Pichia angusta*, and antisense sequence to *gapA* gene	This study
Strains		
SIJ488	*E. coli* K-12 MG1655 Tn7::para-exo-beta-gam; prha-FLP; xylSpm-IsceI	Lab stock
SIJ01	SIJ488, deletion of *frmAB* gene	This study
SIJ02	SIJ01, deletion of *pfkA* and *pfkB* genes	This study
SIJ03	SIJ02, deletion of *sucA* gene	This study
X1	SIJ488, harboring pTrc99a-mdh-das	This study
X2	SIJ01, harboring pTrc99a-mdh-das	This study
X3	SIJ02, harboring pTrc99a-mdh-das	This study
X4	SIJ03, harboring pTrc99a-mdh-das	This study
X5	SIJ03, harboring pTrc99a-mdh-das-antigapA	This study
Ev17	An isolated strain from ALE of strain X5	This study

### 2.2 Plasmids and strains construction

To construct plasmid pTrc99a-mdh-das for the hybrid methanol assimilation pathway, *mdh* gene encoding methanol dehydrogenase from *Acinetobacter garner* and *das* gene encoding dihydroxyacetone synthase from *Pichia angusta* were codon-optimized and synthesized with a consensus RBS (AAGAAGGAGATATAC) under the control of the Trc promoter and inserted into the restriction site of EcoRI/SmaI of pTrc99a. To construct plasmid pTrc99a-mdh-das-antigapA, a fragment containing high-performing MicF M7.4 Hfq binding site (Hoynes-O’Connor and Moon, 2016) with the target binding region (TBR) of *gapA* gene was inserted into plasmid pTrc99a-mdh-das under the control of the Trc promoter. TBR sequence was designed to be complementary to the initiation codon (AUG) and extended 27 nucleotides into the coding region of *gapA* gene. Gibson assembly was used for all plasmid construction following the standard procedure (Gibson et al., 2009).

Gene knockout in *E. coli* SIJ488 was based on the lambda Red recombineering system as described by Jensen (Jensen et al., 2015). All of the primers and synthesized gene sequences used in this study are listed in Supplementary Tables S2, S3.

### 2.3 Medium and culture conditions

Strains were cultivated in M9 minimal medium with 400 mM methanol, 1 g/L yeast extract, and 100 μg/mL ampicillin at 37°C and 200 rpm unless otherwise stated. The M9 minimal medium contains: Na_2_HPO_4_⋅12H_2_O 17.1 g/L, KH_2_PO_4_ 3 g/L, NH_4_Cl 1 g/L, NaCl 0.5 g/L, 2 mM MgSO_4_⋅7H_2_O, 0.1 mM CaCl_2_, trace element solution 250 μL/L. The trace element solution contained FeCl_3_⋅6H_2_O 1.62 g/L, ZnCl_2_ 0.13 g/L, CoCl_2_⋅6H_2_O 0.2 g/L, Na_2_MO_4_⋅2H_2_O 0.2 g/L, CuCl_2_⋅6H_2_O 0.09 g/L, and H_3_BO_3_ 0.05 g/L. If necessary, an amino acid mix solution was additionally added, consisting of (per liter of final culture medium): L-arginine hydrochloride, 72.5 mg; L-cystine 24.0 mg; L-histidine hydrochloride 42 mg; L-isoleucine 52.4 mg; L-leucine 52.4 mg; L-lysine hydrochloride, 126.4 mg; L-methionine,15.1 mg; L-phenylalanine, 33 mg; L-threonine, 47.6 mg; L-tryptophan, 10.2 mg; L-tyrosine, 36 mg; L-valine 46.8 mg. The expression of the hybrid methanol assimilation pathway was induced by adding 0.1 mM isopropyl β-D-1-thiogalactopyranoside (IPTG) initially.

Adaptive laboratory evolution of strain X5 was performed by serial transfers with inoculation of 2% (v/v) cultures every 2–4 days. Medium for evolution was a mixture of M9 minimal medium and Hi-Def Azure (HDA, Teknova) (Bennett et al., 2020b; Chen et al., 2020) with 400 mM methanol, 0.1 mM IPTG, and 100 μg/mL ampicillin. Strain X5 was first cultivated in the 100% HDA medium at 37°C and 200 rpm. After being cultivated to the stationary phase, the cultures were used as seed cultures to inoculate fresh medium containing 75% HDA and 25% M9 minimal medium. From passage 2 to passage 5 (liquid transfer cycles), the ratio of HDA was further reduced from 75% to 30%. From passage 6 to passage 11, cell cultures were continuously transferred in 15% HDA and 85% M9 minimal medium. Starting from passage 12, cell growth was achieved in 5% HDA and 95% M9 minimal medium, and the subsequent evolution was continued with the same medium. At passage 28, a colony was isolated and termed Ev17 strain for further studies.

### 2.4 Analytical methods

The cell concentration was determined at an optical density of 600 nm (OD_600_). The percent increase of biomass caused by methanol was calculated as Eq. 1 (Gonzalez et al., 2018).

$$\frac{{final\;OD}_{600}\left(with\;methanol\right)-{\;final\;OD}_{600}\left(without\;methanol\right)}{final\;{OD}_{600}\left(without\;methanol\right)-initial\;{OD}_{600}\;of\;culture}*100\%$$ (1)

Quantification of methanol concentration was carried out by using High-performance liquid chromatography (HPLC) equipped with an Aminex HPX-87H Column (300 × 7.8 mm) using 5 mM sulfuric acid as the mobile phase with a flow rate of 0.8 mL/min at 65°C. The consumption of methanol has been adjusted by subtracting the evaporation of methanol in the medium without cells. The concentration of formaldehyde was quantified by the Nash reaction (Woolston et al., 2018). 125 μL of cells supernatant was mixed with 125 μL Nash reagent (5 M ammonium acetate, 50 mM acetylacetone). The mixtures were incubated at 37°C for 1 h and measured at 412 nm. Formaldehyde standard solution needs to be prepared fresh daily and the standard curve was in the range from 0 to 100 μM.

### 2.5 ^13^C labeling analysis

To carry out the ^13^C-methanol isotopic analysis for intracellular metabolites and amino acids, cells were cultured in M9 minimal medium with 400 mM ^13^C-methanol and 1 g/L yeast extract for 48 h and prepared according to the protocols described by Long and Antoniewicz (Long and Antoniewicz, 2019). Samples were injected into the UHPLC-Q-Orbitrap liquid chromatography-mass spectrometry system (Thermo Fisher Scientific, United States ). Intracellular metabolites and amino acids were separated on Waters BEH Amide Column (2.1 × 100 mm; 1.7 µm) at 35°C with solvent A (acetonitrile with 0.1% formic acid) and solvent B (deionized water with 0.1% formic acid and 10 mM ammonium acetate) as the mobile phase with a flow rate of 0.3 ml/min. The elution gradient was (% B): 0–5 min at 0%; 5–6 min at 0%–25%; 6–15 min at 25%; 15–16 min at 25%–50%; 16–25 min at 50%; 25–26 min, 0%; 26–30 min, 0%. Mass spectrometry was configured with heated electrospray ionization (HESI) and operated in both positive and negative ion modes in full MS scan mode. Isotopologue fractions (IFs) and average carbon labeling were calculated as Eqs 2, 3 (Keller et al., 2020). ^13^C metabolic tracer analysis was obtained from three biological replicate measurements.

$$Isotopologue\;fractions=\frac{m_{i}}{\sum_{j=0}^{n}m_{j}}$$ (2)

$$Average\;carbon\;labeling=\frac{\sum_{j=0}^{n}m_{j}}{n*\sum_{j=0}^{n}m_{j}}$$ (3)

Here, $m_{i}$ *and* $m_{j}$ are the abundance of a mass isotopologue, *n* is the number of carbon atoms of the metabolite.

### 2.6 Whole-genome sequencing and transcriptomics analysis

DNA re-sequencing and mRNA sequencing were carried out on the Illumina HiSeq instrument by Azenta (Suzhou, China). Cells were cultivated for 48 h in M9 minimal medium with 400 mM methanol and 1 g/L yeast extract. For each sample, 200 μg genomic DNA or 1 μg total RNA was used for library preparation by standard protocols. For transcriptomics analysis, HTSeq (v0.6.1p1) was employed to estimate gene expression levels from the pair-end clean data. Differential expression analysis was by the DESeq2 Bioconductor package which is a model based on the negative binomial distribution. *p*-value of genes were settled <.05 and $\left|\log_{\;2}fold\;changes\right|\geq 1.5$ to detect differential expressed genes. For whole-genome sequencing, BWA (V0.7.17) was used to map clean data to reference genome. The raw datasets can be found in NCBI Bioproject database (accession number: PRJNA896773).

## 3 Results and discussion

### 3.1 Construction of the hybrid methanol assimilation pathway in *E. coli*

**FIGURE 1:**
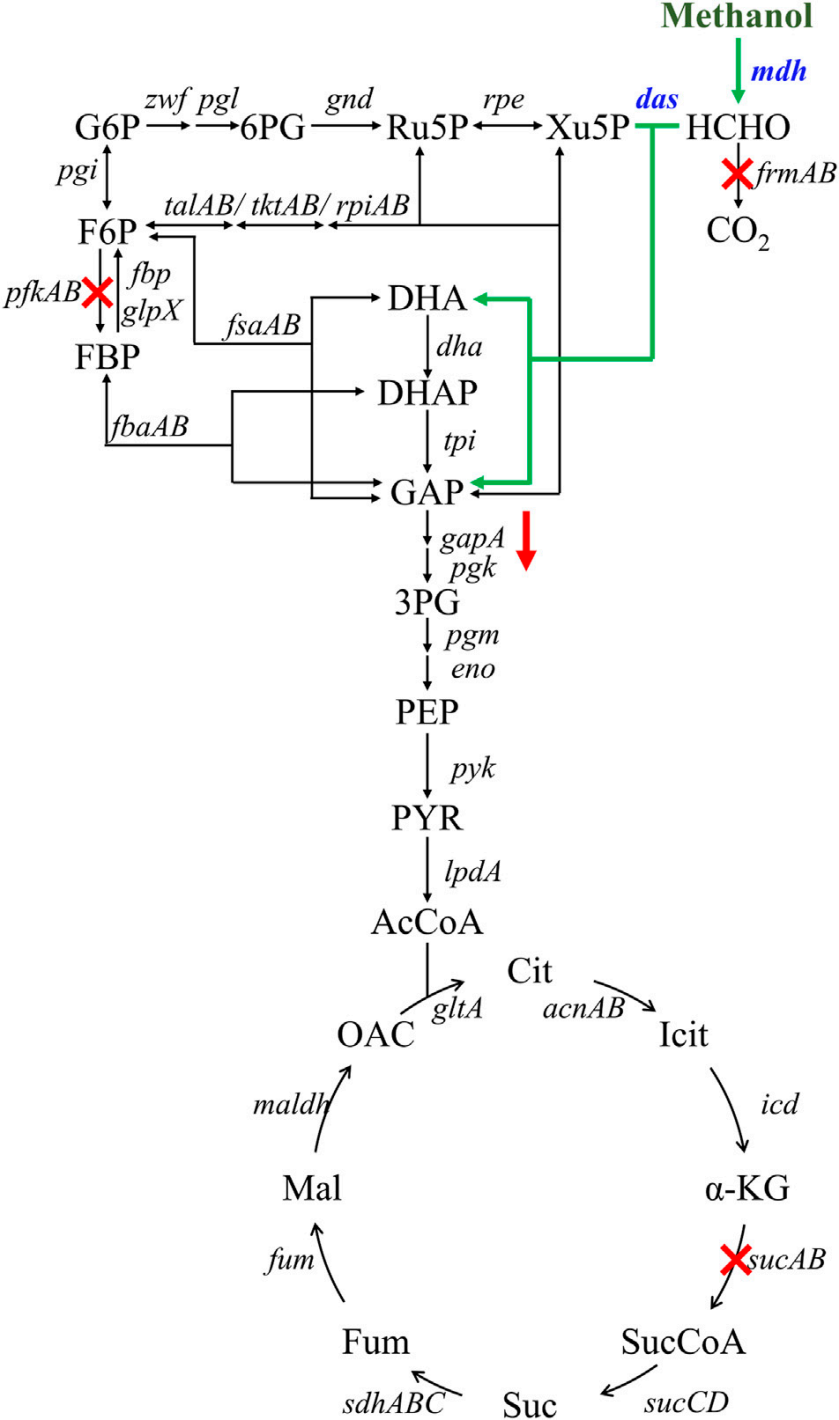
Engineering strategies for improving methanol assimilation based on a hybrid methanol assimilation pathway. The symbol ‘‘X’’ indicates gene deletion and the symbol ‘‘↓’’ indicates down-regulation of gapA gene. Abbreviations: G6P: glucose-6-phosphate; 6PG: 6-phosphogluconate; Ru5P: ribulose-5-phosphate; Xu5P: xylulose 5-phosphate; F6P: fructose-6-phosphate; FBP: fructose-1,6-diphosphate; DHA: dihydroxyacetone; DHAP: dihydroxyacetone phosphate; GAP: glyceraldehyde-3-phosphate; 3PG: glycerate-3-phosphate; PEP: phosphoenolpyruvate; PYR: pyruvate; AcCoA: acetyl-coenzyme A; Cit: citrate; Icit: isocitrate; α-KG: alpha-ketoglutarate; SucCoA: succinyl-coenzyme A; Suc: succinate; Fum: fumarate; Mal: malate; OAC: oxaloacetate.

**FIGURE 2:**
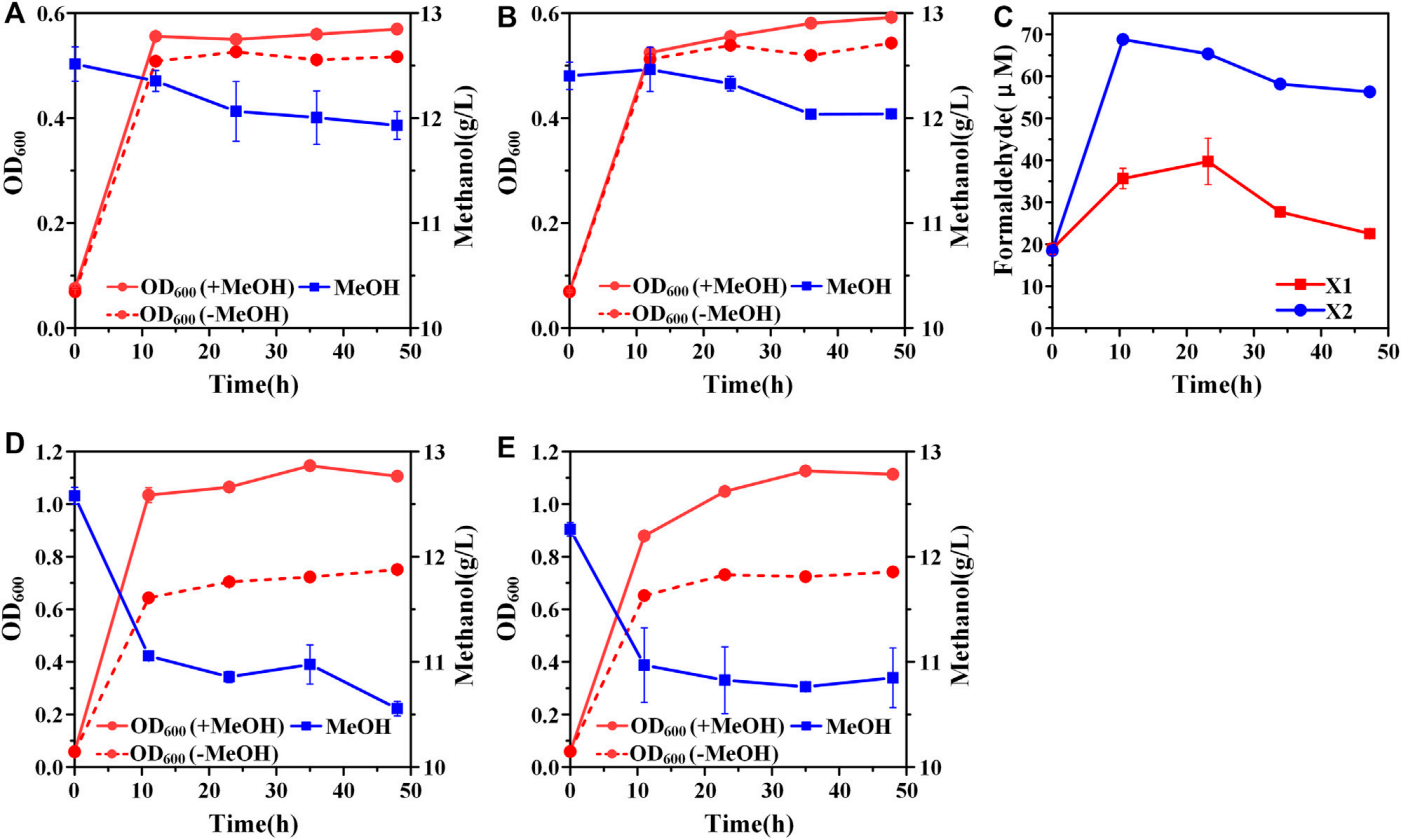
Construction of the hybrid methanol assimilation pathway. **(A)** Growth characteristics of strain X1; **(B)** Growth characteristics of strain X2; **(C)** formaldehyde accumulation. Addition of amino acids mix solution could improve cell growth and methanol consumption of both strain X1 **(D)** and strain X2 **(E)**. Error bars represent standard deviation, *n* = 2.

A hybrid methanol assimilation pathway with prokaryotic NAD-dependent Mdh and eukaryotic dihydroxyacetone synthase (Das) can convert methanol and Xu5P into glyceraldehyde 3-phosphate (GAP) and dihydroxyacetone (DHA) with the generation of NADH. DHA can be further converted into dihydroxyacetone phosphate (DHAP) by the endogenous DHA kinase of *E. coli* (Figure 1). Previously, De Simone et al. showed that a strain expressing *mdh* from *A. garner* and *das* from *P. angusta* exhibited a higher growth rate in minimal medium with methanol and xylose than that with xylose alone (De Simone et al., 2020). In that case, Xu5P was mainly derived from xylose, thus, it was not clear whether the cyclic XuMP pathway would be functional in sugar-free conditions. Thus, we first constructed the same methylotrophic module (*mdh* from *A. garner* and *das* from *P. angusta*) in plasmid pTrc99a and transformed the resulting plasmid pTrc99a-mdh-das into *E. coli* SIJ488 to generate strain X1. When cultured in M9 minimal medium with methanol and 1 g/L yeast extract, strain X1 showed 10.90 ± 0.01% higher final biomass concentration compared to that without methanol, indicating the hybrid methanol assimilation module was functional (Figure 2A). To enhance methanol assimilation, glutathione-dependent formaldehyde dehydrogenase gene (*frmA*) and S-formylglutathione hydrolase gene (*frmB*) were knocked out to reduce formaldehyde dissimilation into CO_2_, generating strain X2. The methanol consumption by strain X2 and the increase of finial biomass concentration by methanol were similar to those of strain X1 (Figure 2B). When cultured with methanol, strains X1 and X2 accumulated 35.66 ± 2.43 µM and 68.78 ± 1.10 µM formaldehyde in 12 h (Figure 2C), suggesting that *mdh* from *A. garner* was successfully expressed in *E. coli*. Blocking the formaldehyde dissimilation pathway in strain X2 significantly increased formaldehyde accumulation but did not improve cell growth and methanol consumption (Table 2), suggesting an imbalance between formaldehyde formation and consumption.

**TABLE 2:** Growth phenotype and methanol consumption of five strains at 48 h.

	X1	X2	X3	X4	X5	Ev17
Methanol consumed (g/L)	0.58 ± 0.03	0.36 ± 0.02	0.25 ± 0.04	0.33 ± 0.04	1.01 ± 0.26	1.63 ± 0.12
OD_**600**_ (+MeOH)	0.57 ± 0.01	0.59 ± 0.00	0.51 ± 0.00	0.38 ± 0.01	0.32 ± 0.00	0.71 ± 0.041
OD_**600**_ (−MeOH)	0.52 ± 0.01	0.54 ± 0.00	0.49 ± 0.00	0.32 ± 0.00	0.26 ± 0.01	0.40 ± 0.01
Increase of biomass caused by methanol	10.90 ± 0.00%	10.60 ± 0.01%	4.70 ± 0.01%	24.00 ± 0.00%	31.60 ± 0.01%	90.9 ± 0.01%
Methanol consumption rate (g/L·OD^−1^)	1.01 ± 0.01	0.61 ± 0.04	0.49 ± 0.00	0.86 ± 0.01	3.16 ± 0.42	2.29 ± 0.13

The percentage increase of biomass caused by methanol was calculated as Eq. 1. The data represent the means ± standard deviations (*n* = 2).

Previous studies showed that the recombinant *E. coli* with Mdh and RuMP pathway cannot efficiently synthesize several amino acids (Bennett et al., 2020a). We hypothesized that the biosynthesis of some endogenous amino acids may also be limited for stains X1 and X2, which may affect cell growth and methanol consumption. When supplemented with an extra amino acid mix solution, strains X1 and X2 showed 52.1 ± 0.04% and 54.4 ± 0.05% increases of final biomass concentration by methanol respectively (Figures 2D, E). Moreover, the consumption of methanol by strains X1 and X2 was increased to 2.02 ± 0.01 g/L and 1.41 ± 0.22 g/L, which were both over 2-fold higher than those without amino acid mix solution (Figures 2D, E). The significant increase of methanol consumption and cell growth benefit by methanol confirmed that some amino acids or their intermediates cannot be efficiently synthesized in strains X1 and X2. It should be noted that the consumption of methanol by strain X2 was still lower than X1 even with the addition of amino acids mix solution. We hypothesized that it was due to the cyclic XuMP pathway was not active and the regeneration of Xu5P was not sufficient for formaldehyde assimilation. Thus, we decided to further improve the efficiency of the cyclic XuMP pathway by rational optimization.

### 3.2 Rational design of the methylotrophic chassis

**FIGURE 3:**
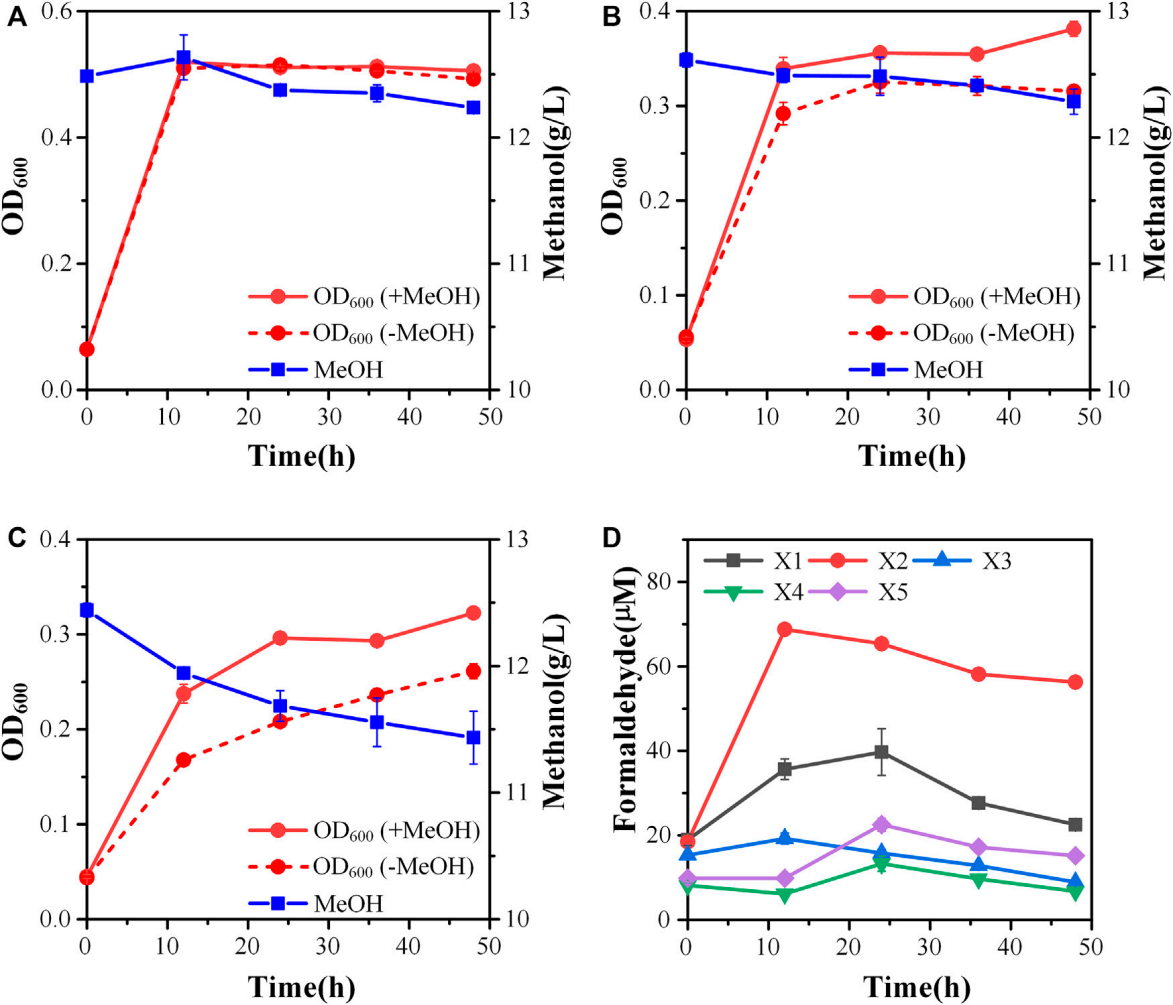
Engineering of the methylotrophic chassis to improve methanol assimilation. **(A)** Growth characteristics of strain X3; **(B)** Growth characteristics of strain X4; **(C)** Growth characteristics of strain X5; **(D)** formaldehyde accumulation. Error bars represent standard deviation, *n* = 2.

To increase the efficiency of methanol assimilation, we tried to divert the metabolic flux toward the cyclic XuMP pathway. GAP and fructose-6-phosphate (F6P) are two key metabolic nodes linking the active XuMP cycle with the glycolysis pathway and pentose phosphate pathway. Balancing the supply and consumption of GAP and F6P is highly important for the functional XuMP cycle as well as for Xu5P regeneration and biosynthesis. Phosphofructokinase is a key enzyme catalyzing the irreversible phosphorylation of F6P to fructose-1,6-bisphosphate, diverting the intracellular pool of fructose-6-phosphate to the glycolysis pathway (Figure 1). Previous studies showed that the high activity of phosphofructokinase in *E. coli* tend to destabilize the cyclic RuMP system or CO_2_ assimilation cycle (Antonovsky et al., 2016; Gleizer et al., 2019). Thus, we first tried to reduce the diversion of flux away from the XuMP cycle by knocking out *pfkAB* genes encoding 6-phosphofructokinase of strain X2. The resulting strain X3 did not show improved methanol consumption or cell growth on methanol compared to strain X2 (Figure 3A), indicating that deleting *pfkAB* genes alone is not sufficient to increase methanol assimilation. However, the accumulation of formaldehyde by strain X3 was significantly reduced compared to strain X2 (Figure 3D), suggesting an improved balance of formaldehyde formation and consumption.

We then tried to further increase the supply of GAP to support the functional XuMP cycle in strain X3. For the aerobic cultivation of *E. coli* on sugars, GAP is mainly diverted to the lower glycolysis pathway and then mainly consumed *via* TCA cycle. Natural methylotrophs such as *Methylobacillus flagellatus* and *B. methanolicus* often contain an incomplete or less active TCA cycle which may be beneficial for retaining a high flux to formaldehyde assimilation pathway (e.g., RuMP pathway) and reducing NADH generation (Müller et al., 2015). NADH is a kinetic inhibitor of methanol dehydrogenase and high generation of NADH *via* the TCA cycle may disturb the redox balance and reduce methanol assimilation (Meyer et al., 2018). Reduced metabolic flux toward the TCA cycle was also observed for synthetic methylotrophs of *E. coli* (Meyer et al., 2018; Chen et al., 2020). Thus, we knocked out the *sucA* gene encoding E1 subunit of the α-ketoglutarate dehydrogenase to down-regulate the TCA cycle of strain X3, generating strain X4. Strain X4 showed a lower final cell density than strain X3, however, methanol consumption by strain X4 was increased (Figure 3B). Especially, the percent increase of biomass by methanol was increased to 24.00 ± 0.01% (Table 2), indicating that deletion of *sucA* gene to reduce the TCA cycle activity was beneficial for methanol assimilation. To further increase methanol assimilation, we set out to adjust the flux distribution at GAP node. Previous studies showed that the high activity of glyceraldehyde 3-phosphate dehydrogenase would strongly divert the metabolic flux to the glycolysis pathway, destabilizing the cyclic formaldehyde assimilation pathway (Meyer et al., 2018; Chen et al., 2020). To reduce the activity of glyceraldehyde 3-phosphate dehydrogenase, we introduced the antisense RNA to inhibit the expression of *gapA* gene of strain X4, giving strain X5. With this modification, methanol consumption by strain X5 was increased to 1.01 ± 0.26 g/L, which was over 2-fold higher than strain X4 (Figure 3C). Moreover, the percent increase of biomass by methanol was increased to 31.6 ± 0.01% (Table 2). Although strain X5 had the highest methanol consumption rate among all of the engineered strains, the low accumulation of formaldehyde during the cultivation indicated an increased formaldehyde consumption *via* the assimilation pathway (Figure 3D). Thus, reducing the activity of glyceraldehyde 3-phosphate dehydrogenase was also highly important for the functional cyclic XuMP pathway.

### 3.3 Adaptive laboratory evolution

**FIGURE 4:**
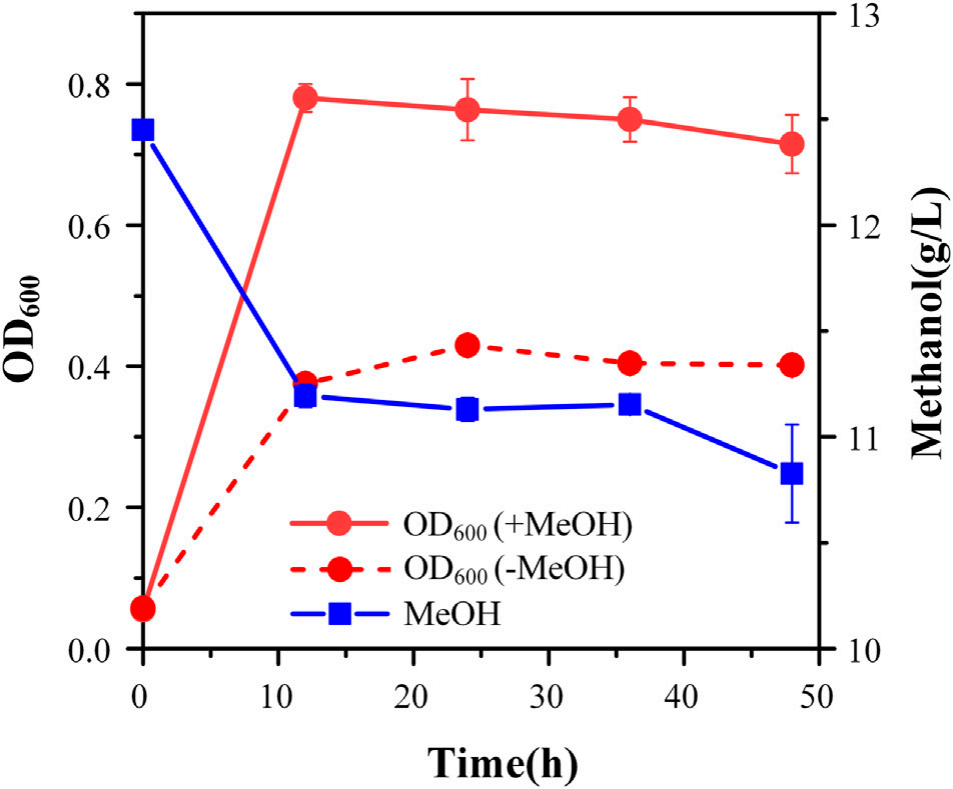
Cell growth and methanol consumption of strain Ev17. Error bars represent standard deviation, *n* = 2.

**FIGURE 5:**
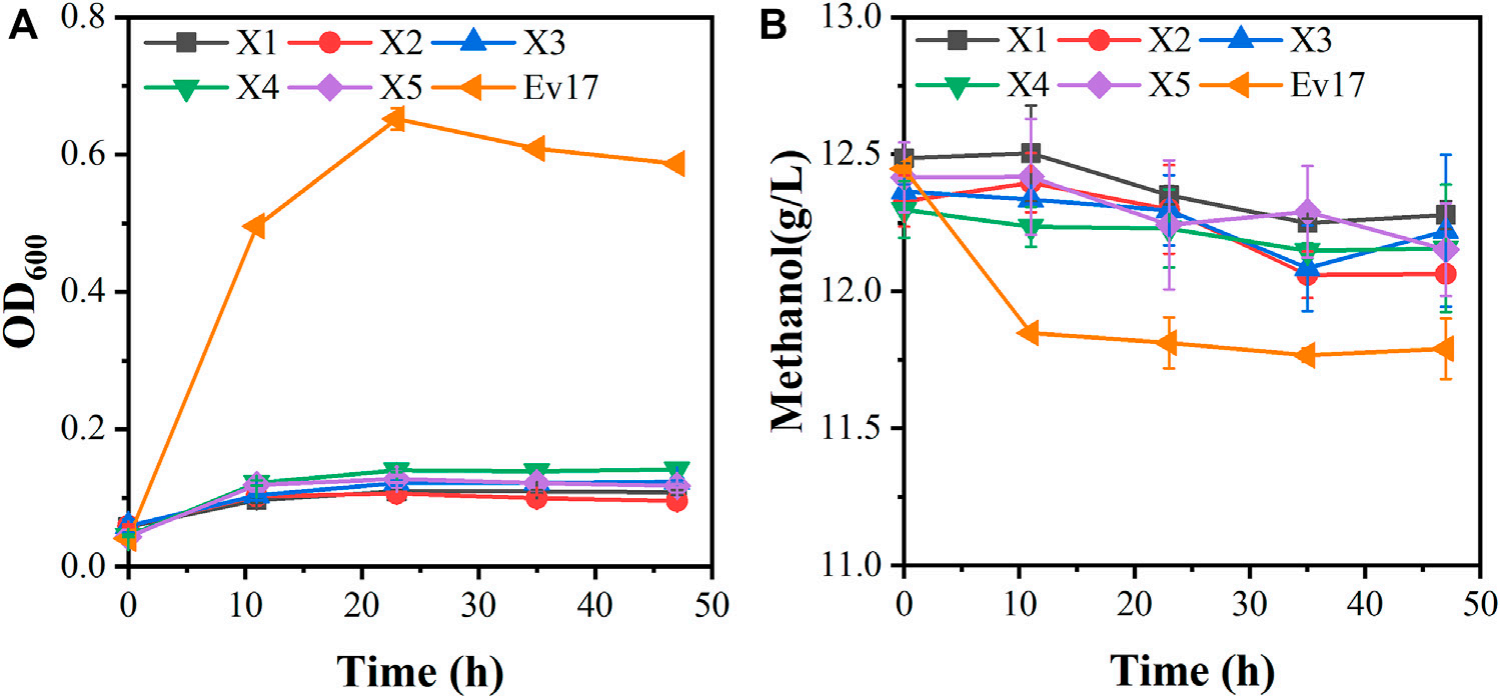
Growth characteristics of engineered strains on methanol with 0.1 g/L yeast extract. **(A)** Cell growth; **(B)** methanol consumption. Error bars represent standard deviation, *n* = 2.

Although strain X5 showed improved methanol utilization, the final cell density was still lower than strain X1. To promote cell growth of strain X5 on methanol, ALE was performed. Strain X5 was first cultivated in Hi-Def Azure (HDA) medium, a semi-minimal medium containing amino acids (Chen et al., 2020). The percent of HDA was gradually reduced and replaced by M9 minimal medium during the evolution. After 12 passages, the culture can grow on methanol with 5% HDA while strain X5 cannot grow on methanol with less than 15% HDA (Supplementary Figure S1). After 28 passages, a best growing single colony in the medium with 5% HDA was isolated and termed Ev17. When cultured in M9 minimal medium with methanol and 1 g/L yeast extract, the final cell density (OD_600_) of strain Ev17 reached 0.71 ± 0.04 and the percent increase of biomass by methanol achieved 90.9 ± 0.01%, which was 1.8-fold higher than that of strain X5 (Figure 4). Moreover, strain Ev17 consumed 1.62 ± 0.23 g/L methanol, which was 60.4% higher than strain X5. Especially, when cultured with methanol and 0.1 g/L yeast extract, strain Ev17 achieved OD_600_ 0.65 ± 0.016 and consumed 0.66 ± 0.11 g/L methanol while other unevolved strains only showed marginal methanol consumption and cell growth (Figure 5), indicating that methanol assimilation was significantly improved by ALE.

### 3.4 ^13^C-methanol incorporation into intracellular metabolites and amino acids

**FIGURE 6:**
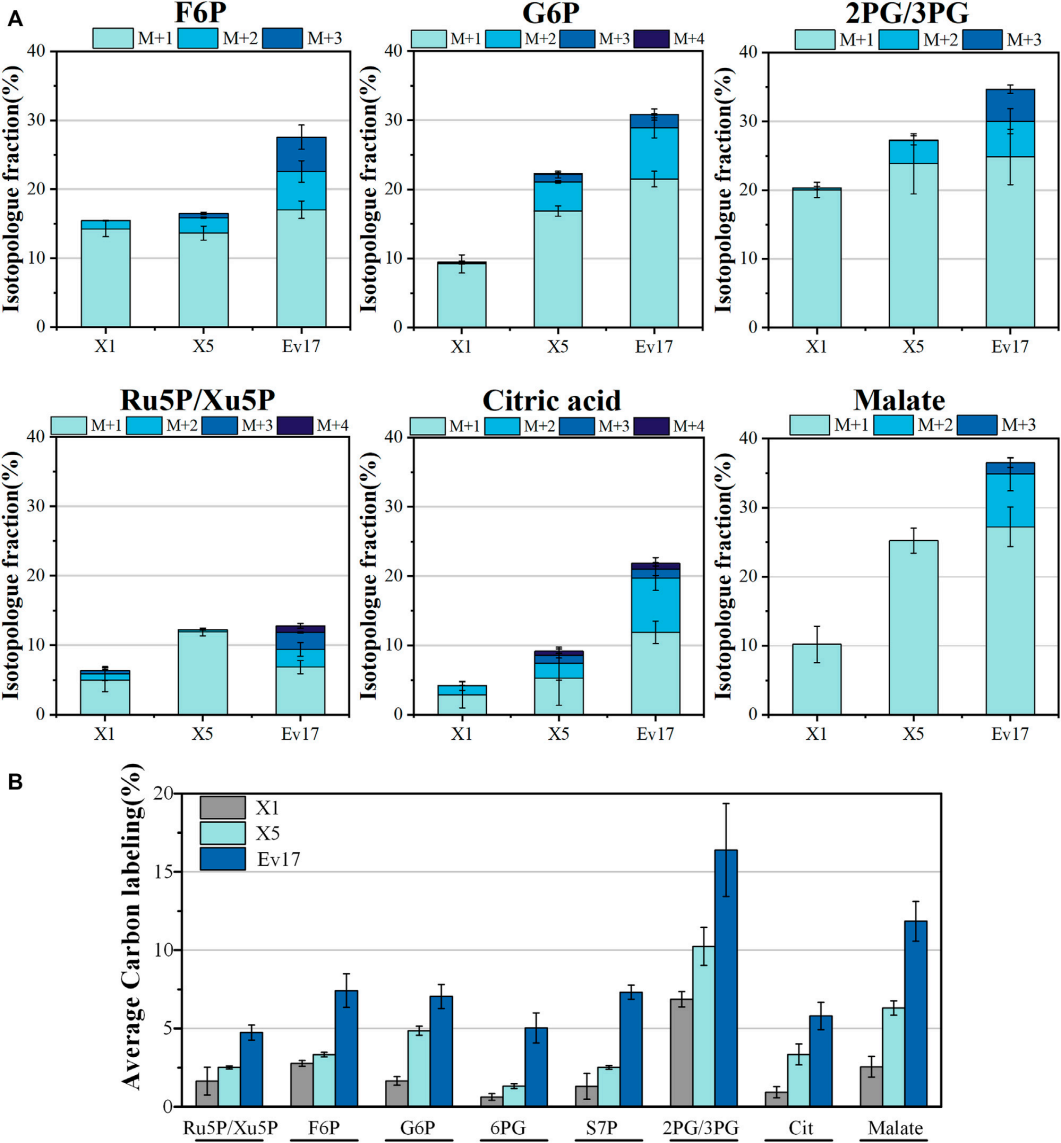
Isotopologue fraction (% ^13^C) **(A)** and average carbon labeling of intracellular metabolites **(B)** in strain X1, X5, Ev17. Error bars represent standard deviation, *n* = 2. Abbreviations: F6P: fructose-6-phosphate; G6P: glucose-6-phosphate; 2/3PG: glycerate-3-phosphate/glycerate-2-phosphate; Ru5P/Xu5P: ribulose-5-phosphate/xylulose 5-phosphate; 6PG: 6-phosphogluconate; S7P: sedoheptulose-7-phosphate; Cit: citrate.

**FIGURE 7:**
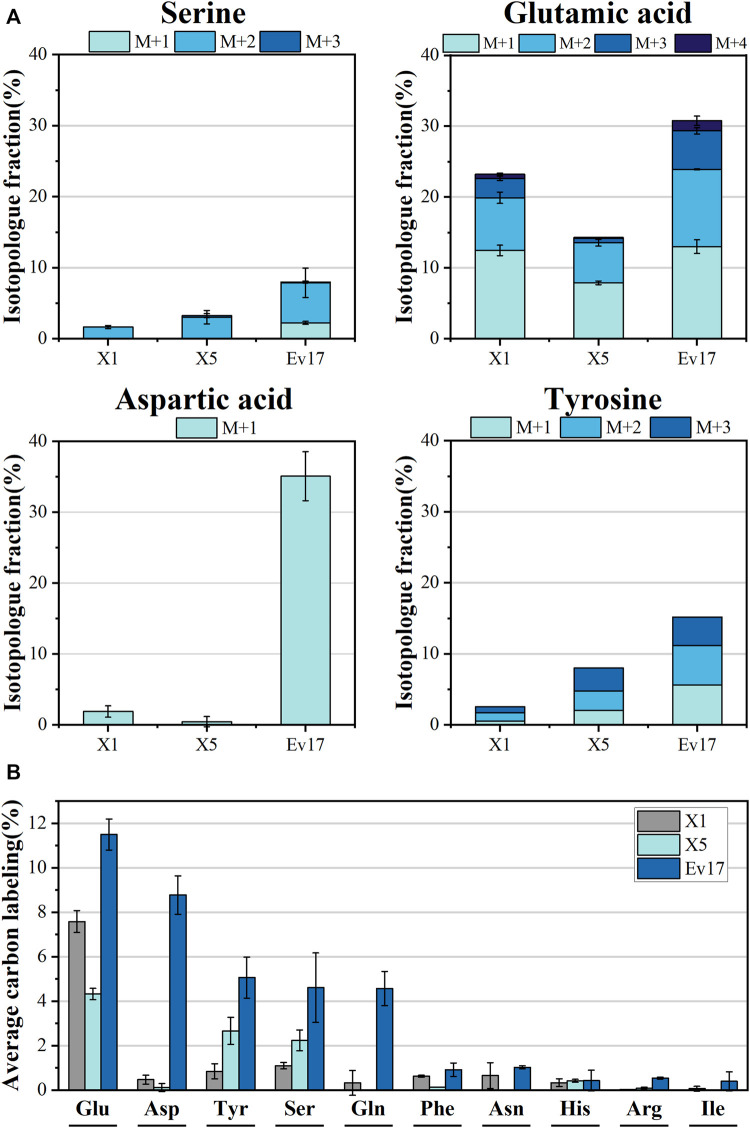
Isotopologue fraction (% ^13^C) **(A)** and average carbon labeling **(B)** of amino acids in strain X1, X5, and Ev17. Error bars represent standard deviation, *n* = 2.

To further evaluate the impact of rational design and ALE on methanol assimilation, ^13^C-labeling analysis was performed with strains X1, X5, and Ev17. We cultured strains with ^13^C-methanol and 1 g/L yeast extract and measured the intracellular metabolites and amino acids using liquid chromatography-mass spectrometry (Figures 6, 7). Introducing the hybrid methanol assimilation module alone in strain X1 resulted in 6.87 ± 0.48% ^13^C-labeling of 2-phosphoglycerate/3-phosphoglycerate (2PG/3PG) and low but significant ^13^C-labeling of several important metabolites in glycolysis, TCA cycle, and pentose phosphate pathway (Figure 6). However, most of these labeled metabolites contained only one ^13^C atom (M1). Significant improvement of ^13^C-labeling of all measured metabolites was observed in both strains X5 and Ev17. For example, the average ^13^C-labeling of 2PG/3PG in strains X5 and Ev17 was increased by 49.1% and 138.6% compared with strain X1. Especially, the increase of ^13^C-labeling of Ru5P/Xu5P in strain X5 demonstrated the improved XuMP cycle by rational engineering. Strain X5 also exhibited the labeling of several metabolites with more than one ^13^C atom, such as 2PG/3PG, glucose-6-phosphate (G6P) and citric acid, which were caused by XuMP cycle running more than one time. All of the measured metabolites in strain Ev17 showed higher average ^13^C-labeling and had more than one ^13^C atom, confirming the significant improvement of methanol assimilation *via* ALE.

To confirm that methanol was involved in biosynthetic pathways, ^13^C-labeling analysis of intracellular amino acids was also carried out (Figure 7). Compared to strain X1, the significant increase in average labeling of glutamic acid (7.58 ± 0.50%), L-tyrosine (2.66 ± 0.61%) and L-serine (2.23 ± 0.47%) was detected in strain X5. Strain Ev17 displayed significant ^13^C-methanol incorporation into glutamic acid (11.50 ± 0.70%), aspartic acid (8.77 ± 0.86%), tyrosine (5.06 ± 0.92%), serine (4.61 ± 1.57%), and glutamine (4.57 ± 0.77%). These improvements supported that our strategies enhanced the biosynthesis of amino acids from methanol.

### 3.5 Transcriptomics analysis and genome sequencing

**FIGURE 8:**
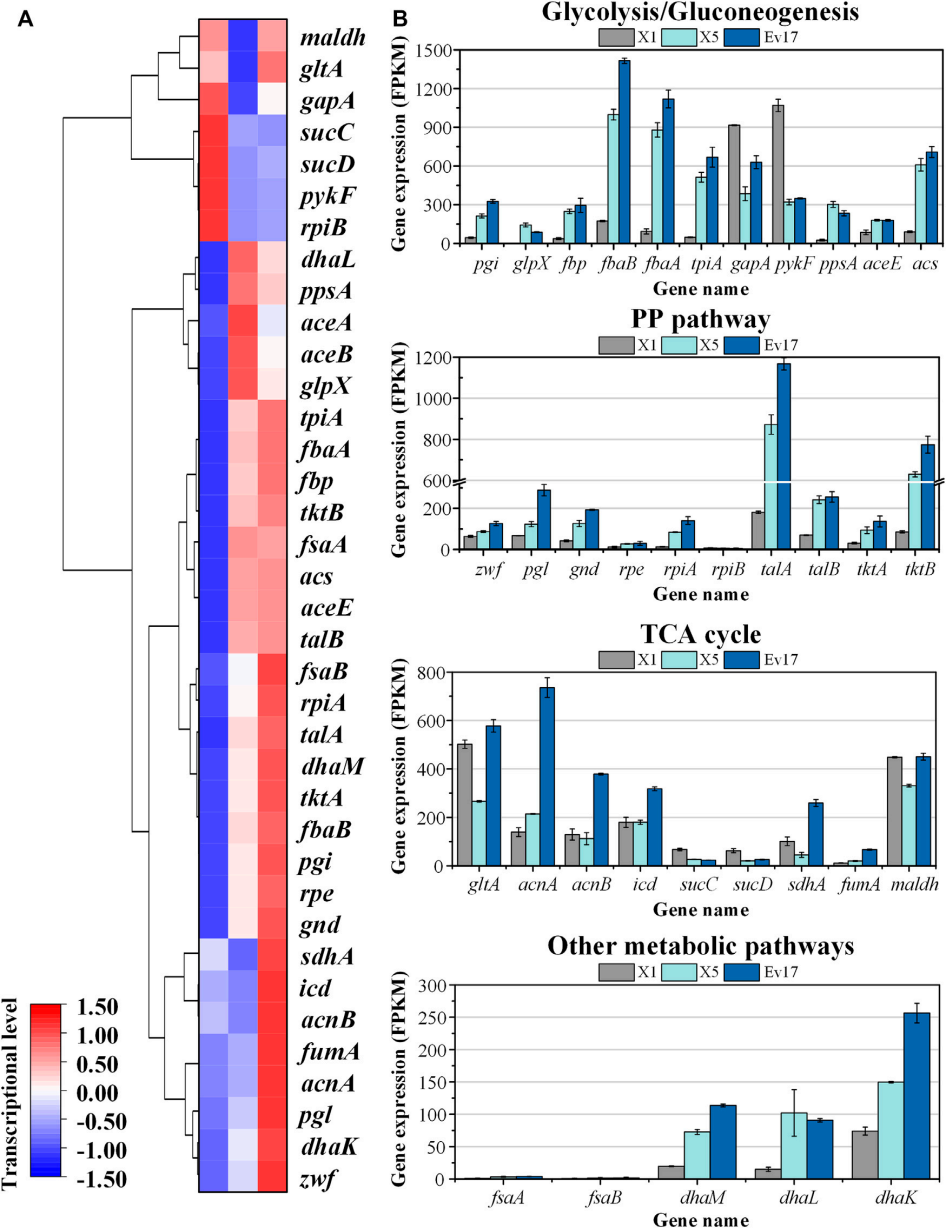
Transcriptomics analysis of strain X1, X5, and Ev17. **(A)** Heat map and hierarchical clustering of differentially expressed genes. **(B)** Gene expression of glycolysis/gluconeogenesis pathways, TCA cycle, PP pathway and other metabolic pathways of strain X1, X5, and Ev17. The transcriptional level (FPKM) has been normalized (*n* = 2).

To analyze the change of global genes expression, transcriptomics analysis of strains X1, X5, and Ev17 was further performed (Figure 8). Comparing with strain X1, most of the genes in TCA cycle, including *maldh*, *gltA, sucCD, sdhA*, were down-regulated in strain X5, indicating that deletion of *sucA* successfully reduced the activity of TCA cycle. Owing to the introduction of antisense RNA, the expression of *gapA* and *pyk* (encoding pyruvate kinase) in strain X5 were also down-regulated, demonstrating that reducing the activities of glycolysis and TCA cycle is beneficial for the higher methanol assimilation (Figure 8A). Most of genes related to DHA and GAP assimilation, such as *fbaAB* (encoding fructose-bisphosphate aldolase), *fbp* (encoding fructose-bisphosphatase), and the *dhaLMK* operon (encoding dihydroxyacetone kinase), were significantly up-regulated in strain X5 and Ev17. However, the fructose-6-phosphate aldolase (FSA) pathway encoded by *fsaAB* operon in all three strains was not transcriptionally activated (Figure 8B). These results indicated that Xu5P regeneration was successfully enhanced mainly assimilated *via* the dihydroxyacetone kinase pathway which is consistent with a previous study by (Peiro et al., 2019). Besides, the up-regulation of *talAB operon* (encoding transaldolase), *tktAB* operon (encoding transketolase) in strain X5 and Ev17 also contributed to the Xu5P regeneration (Figure 8B).

We further performed the Whole Genome Sequencing (WGS) analysis of stain Ev17. No mutations were found in the plasmid and all mutations in the genome were listed in Supplementary Table S4. Several mutations on the transcriptional regulators such as *narL*, *gntR*, and *iclR* were identified. The mutations of global transcriptional factors may play important roles in methanol metabolism and cell growth. In addition, mutations on *gltA* and *aceK* genes were also identified, which probably alter the activity of glyoxylate pathway (due to the knockout of *sucA* gene). Mutations on genes related to amino acids biosynthesis, such as *glnE* (encoding glutamine synthetase), *sdaB* (encoding serine deaminase), and *asnB* (encoding asparaginase) were also identified. The function of these mutations and their relations to methanol assimilation should be clarified in the future.

## 4 Conclusion

Developing a synthetic methylotrophy for C1 utilization is a big challenge for synthetic biology. In this study, we attempted to engineer a synthetic *Escherichia coli* based on a hybrid methanol assimilation pathway. The final engineered strain was successfully established by rational design and evolution and has a methanol-dependent growth phenotype with only 0.1 g/L yeast extract as co-substrate. The results showed that a combination of knocking out *pfkAB*, *sucA*, and introducing antisense RNA of *gapA* was efficient for methanol utilization. Further improvement of cell growth by ALE enabled the engineered strain to form 90.9 ± 0.01% increase of biomass by methanol and remarkable incorporation of ^13^C-methanol into intracellular metabolites and amino acids, especially 2PG/3PG (16.39 ± 2.97%), citric acid (5.80 ± 0.87%) and glutamic acid (11.50 ± 0.70%) which were all improved over 2.5-fold than the initial strain. Notably, more than one ^13^C atom of metabolites was detected, suggesting an effective XuMP cycle after engineering. Transcriptomics analysis demonstrated that FBA, FBP, and DAK pathways served as the key reactions for Xu5P regeneration while the FSA pathway was not transcriptionally activated. Since the of FSA variant of XuMP pathway is more energy-efficient (Supplementary Table S1), it is possible to overexpress the corresponding *fsaAB* genes to increase the pathway efficiency in the future. Further combination of rational design and ALE of strain Ev17 can be carried out in the future to develop synthetic methylotrophy that grow solely on methanol.

## Data Availability

The datasets presented in this study can be found in online repositories. The names of the repository/repositories and accession number(s) can be found in the article/Supplementary Material.
