# Supplementary figures and images for: Engineering and adaptive laboratory evolution of Escherichia coli for improving methanol utilization based on a hybrid methanol assimilation pathway
Source: Front Bioeng Biotechnol. 2023 Jan 10;10:1089639. doi: 10.3389/fbioe.2022.1089639 (PMC9871363; doi:10.3389/fbioe.2022.1089639)

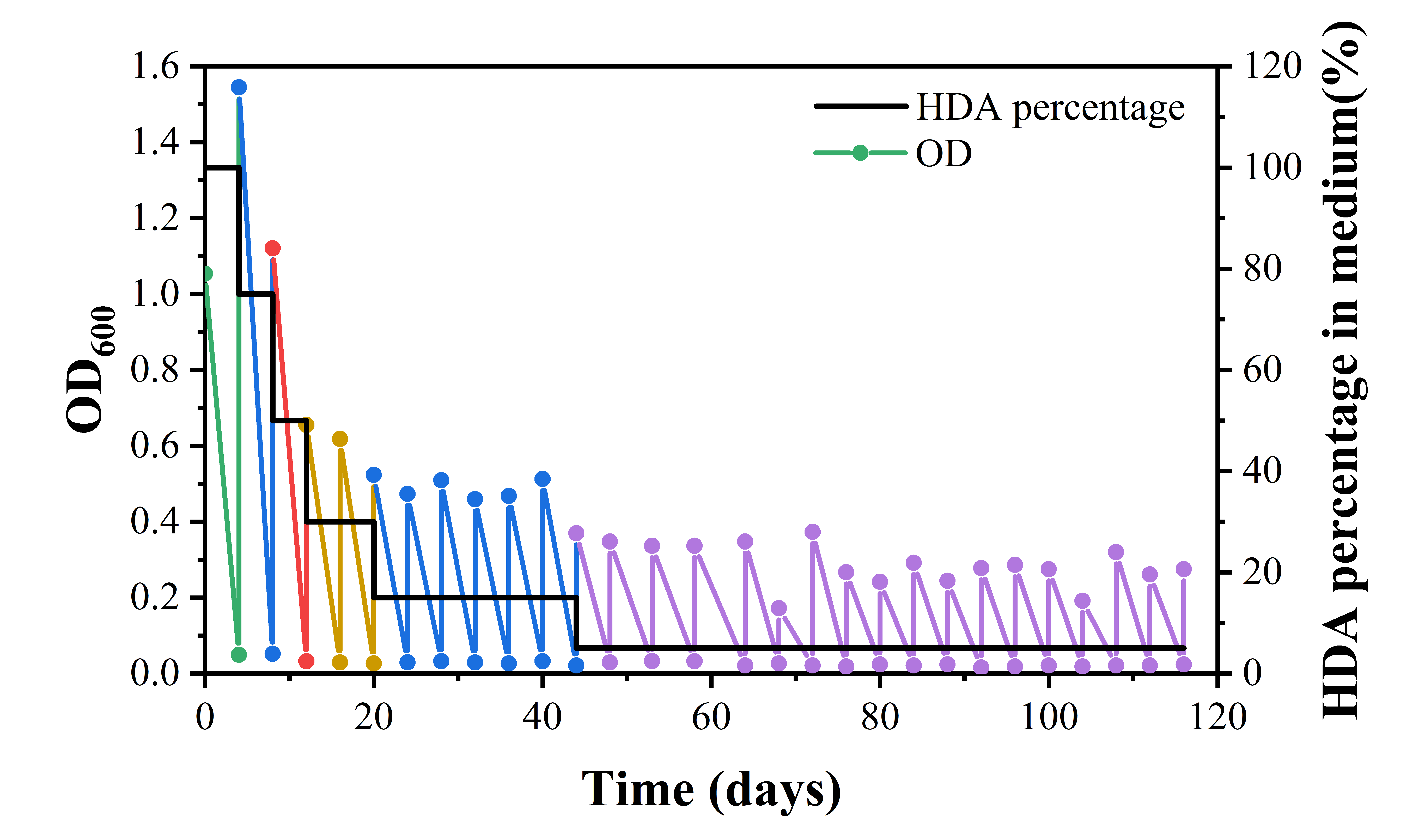

Supplement: Supplementary file 1 [file Image1.TIF]
