# Supplementary material for: Engineering and adaptive laboratory evolution of Escherichia coli for improving methanol utilization based on a hybrid methanol assimilation pathway
Source: Front Bioeng Biotechnol. 2023 Jan 10;10:1089639. doi: 10.3389/fbioe.2022.1089639 (PMC9871363; doi:10.3389/fbioe.2022.1089639)
Supplement: Supplementary file 2 [file DataSheet1.pdf]

Supplementary Material

Engineering and adaptive laboratory evolution of *Escherichia coli* for improving methanol utilization based on a hybrid methanol assimilation pathway

Qing Sun[1], Dehua Liu[1,2,3], Zhen Chen[1,2,3*]

[1] Key Laboratory of Industrial Biocatalysis (Ministry of Education), Department of Chemical Engineering, Tsinghua University, Beijing, China

[2] Tsinghua Innovation Center in Dongguan, Dongguan, China

[3] Center for Synthetic and Systems Biology, Tsinghua University, Beijing, China

*** Correspondence:**

Zhen Chen,

zhenchen2013@mail.tsinghua.edu.cn

1 Supplementary Figures

Fig S1. Adaptive laboratory evolution process of strain X5. Percentage of HDA in final medium was presented by the black lines.

2 Supplementary Tables

Table S1. Comparison of ATP efficiency between different methanol assimilation pathways

Table S2. Primers used in this study

Table S3. Synthesized genes and their optimized sequences used in this study

Table S4. Mutations in the evolved Ev17 strain relative to the parent strain

Table S1. Comparison of ATP efficiency between different methanol assimilation pathways

Pathway	Representative microorganisms	Pathway stoichiometry	Key enzyme
RuMP	*Bacillus methanolicus*	3Methanol+4NAD^++ADP→ Pyruvate+4NADH+ATP (TA)	Mdh, Hps, Phi
XuMP	*Pichia pastoris*	3Methanol+3O_2+NAD^++ATP→ Pyruvate+3H_2O_2+NADH+ADP	AOX, Das
Serine cycle	*Methylobacterium extorquens*	2Methanol+ CO_2+2PQQ+FP +2NADH+ 2ATP→ Pyruvate+2$PQQH_2$+FPH_2+2NAD^+ +2ADP	SHMT
rGly	Engineered *E. coli*	2Methanol+CO_2+3NAD^+ +2NADPH+2ATP→ Pyruvate+3NADH+2$NADP^+$+ 2ADP	Mdh, Ftfl, Fch,MdtA
Hybrid XuMP pathway	Engineered *E. coli*	3Methanol+4NAD^++ADP→ Pyruvate+4NADH+ATP	Mdh, Das, FSA
		3Methanol+4NAD^++ATP→ Pyruvate+4NADH+ADP	Mdh, Das, FBA

Table S2. Primers used in this study

Primer name	Sequence (5′– 3′)
**pTrc99a-mdh-das construction**	
mdh-das-Frag.F	gcggataacaatttcacacaggaaacagaccatggaattcaagaaggagatatacatggcgttcagaac
mdh-das-Frag.R	Agcttgcatgcctgcaggtcgactctagaggatccccgggttaaagtttattcactttgtcgtggtttggct
**pTrc99a-mdh-das-antigapA construction**	
backbone-F	gagaatccacccggggatcctctagagtcg
backbone-R	Cattatacgagccggatgattaattgtcaattaaagtttattcactttgtcgtcgtggtttggc
Fragment-F	Ttgacaattaatcatccggctcgtataatgaaaaccgttgatacctactttgatagtcatcgtcccgcaagga tgcg
Fragment-R	gactctagaggatccccgggtggattctcaccaataaaaaacgcccg
**frmAB_KO_fragment construction**	
frmAB_KO-F	Acgccagcaccagcgcgcccagcccgcccatagaatgaccagagatagacgtgtaggctggagctgc ttc
frmAB_KO-R	cgtgaccaccggtattggcgcggtacacaacacagctaaagtccagccagctgatcagtgataagctgtc aaacatgag
**pfkA_KO_fragment construction**	
pfkA_KO-F	Caatggccgcagccaacgtcagatctccacaataacggcccatcacttccgtgtaggctggagctgcttc

pfkA_KO-R	agacttccggcaacagatttcattttgcattccaaagttcagaggtagtcctgatcagtgataagctgtcaaacatgag
**pfkB_KO_fragment construction**	
pfkB_KO-F	actttccgctgattcggtgccagactgaaatcagcctataggaggaaatgctgatcagtgataagctgtcaaacatgag
pfkB_KO-R	Ccaactcgatgttaccaattgccagtgctgcacttaacgcttcgccagaagtgtaggctggagctgcttc
**sucA_KO_fragment construction**	
sucA_KO-F	ggtagtatccacggcgaagtaagcataaaaaagatgcttaagggatcacgctgatcagtgataagctgtcaaacatgag
sucA_KO-R	Ccaacttcataaccacgcgctttcgacatgttcagggtttcctgaaccacgtgtaggctggagctgcttc

Table S3. Synthesized genes and their optimized sequences used in this study

Gene name	Sequence (5′– 3′)
mdh	ATGGCGTTCAAGAACCTGGCGGATCAGACTAATGGTTTCTACATCCCGTG<br>CGTCAGCTTGTTCGGTCCTGGGTGTGCAAAAGAAGTGGGGGCCAAAGCAC<br>AAAACCTTGGGGCTAAAAAAGCGTTGATCGTCACCGATGCAGGGCTTTTT<br>AAATTCGGAGTGGCAGACATCATCGTAGGGTACCTTAAAGATGCGGGAG<br>TCGACAGTCATGTGTTTCCGGGGGCAGAACCCAATCCCACGGACATTAAT<br>GTATTGAATGGGGTTCAGGCCTATAATGATAATGGATGCGATTTTATTGTT<br>TCTCTGGGTGGAGGCAGCTCACACGACTGCGCTAAGGGCATTGGGCTGGT<br>GACGGCTGGTGGGGGTAACATTCGCGATTATGAGGGTATTGACAAATCGT<br>CAGTCCCCATGACACCCTTAATTGCGATCAATACGACCGCTGGAACAGCC<br>TCGGAAATGACCCGTTTTTGTATTATCACAAATACAGATACCCACGTTAA<br>GATGGCAATCGTGGATTGGCGCTGTACTCCTTTGGTCGCTATTGACGACC<br>CGAAATTGATGATTGCTAAACCGGCTGCCCTGACTGCAGCTACTGGCATG<br>GATGCGCTTACACATGCGGTAGAAGCGTACGTGTCGACTGCAGCCAACCC<br>CATTACAGATGCATGCGCTGAAAAGGCGATCAGCATGATTTCTGAGTGGT<br>TGAGTTCAGCAGTAGCAAACGGGGAAAACATCGAAGCGCGCGACGCTAT<br>GGCTTACGCTCAGTATCTTGCGGGTATGGCGTTTAATAATGCGTCGTTAG<br>GATATGTGCACGCCATGGCCCACCAGCTGGGTGGGTTTTACAATTTACCT<br>CACGGTGTATGTAACGCCATCCTGCTGCCACACGTATGCGAGTTCAATCT<br>TATTGCGTGCCCCGATCGTTTTGCGAAGATTGCACAGCTGATGGGCGTAG<br>ATACAACAGGTATGACGGTAACTGAGGCGGGTTACGAGGCTATTGCAGC<br>GATTCGCGAGCTTTCAGCCAGTATTGGGATTCCGAGCGGGCTGACCGAAC<br>TGGGGGTAAAAGCCGCAGACCATGCTGTAATGACGTCTAACGCGCAAAA<br>AGATGCTTGCATGCTGACAAATCCTCGCAAAGCGACTGACGCGCAGGTGA<br>TTGCTATTTTCGAAGCAGCTATGTAA
das	ATGTCCATGCGCATTCCCAAAGCGGCGAGCGTAAACGACGAGCAGCATC<br>AACGCATTATCAAATATGGCCGCGCCTTGGTCCTGGACATTGTTGAACAG<br>TACGGGGGAGGGCATCCCGGATCGGCCATGGGCGCGATGGCAATCGGCA<br>TTGCATTATGGAAGTATACACTTAAGTACGCTCCTAATGATCCAAATTACT<br>TCAATCGTGATCGCTTCGTTTTGAGTAATGGTCACGTTTGTCTTTTTCAGT<br>ACATCTTCCAGCACTTGTATGGGTTAAAGTCCATGACGATGGCGCAGTTA

AAGTCTTACCACTCGAATGACTTTCATTCATTATGTCCTGGCCACCCGGAG
ATCGAACACGATGCGGTTGAGGTCACTACCGGACCGCTGGGGCAGGGAA
TCTCTAACTCGGTTGGTTTGGCTATCGCCACCAAGAACCTTGCCGCGACCT
ACAACAAACCAGGCTTCGATATCATTACTAATAAAGTTTACTGTATGGTT
GGAGATGCATGTTTACAAGAAGGGCCTGCTCTGGAAAGTATCAGTTTGGC
TGGACACATGGGGTTGGATAATTTAATTGTCCTTTACGACAACAATCAAG
TATGCTGCGATGGCTCGGTCGACATCGCAAATACCGAGGATATCTCGGCT
AAATTTAAGGCGTGCAATTGGAATGTCATCGAGGTTGAAAATGCCAGTGA
GGACGTTGCAACCATCGTCAAGGCGCTGGAATATGCACAGGCTGAGAAG
CACCGCCCGACTTTAATTAATTGTCGTACTGTTATCGGTAGTGGGGCAGC
ATTTGAGAACCACTGTGCAGCTCATGGGAACGCTTTAGGCGAAGACGGG
GTACGCGAGTTAAAAATTAAATACGGAATGAACCCGGCGCAAAAATTTT
ATATTCCGCAGGATGTATATGACTTTTTCAAAGAGAAGCCAGCCGAAGGT
GACAAGCTGGTTGCCGAGTGGAAAAGTCTTGTGGCCAAGTACGTGAAGG
CATACCCGGAAGAGGGTCAAGAATTCCTTGCCCGTATGCGCGGCGAATTG
CCCAAAAACTGGAAGTCGTTTCTTCCCCAACAAGAGTTTACAGGAGATGC
TCCCACACGCGCCGCTGCCCGTGAGTTGGTTCGCGCCCTTGGTCAAAATT
GTAAGTCTGTAATCGCTGGATGTGCCGACTTGTCCGTTTCGGTGAATTTAC
AGTGGCCTGGCGTTAAGTACTTCATGGATCCCTCATTATCCACGCAGTGC
GGGCTTTCCGGAGATTATTCGGGTCGTTATATCGAGTACGGTATTCGCGA
ACATGCGATGTGTGCGATCGCTAACGGATTGGCAGCCTATAATAAAGGGA
CGTTTCTGCCCATCACGTCGACCTTCTTCATGTTCTATTTGTACGCTGCGC
CTGCCATTCGTATGGCAGGCCTTCAAGAGTTAAAGGCTATCCATATCGGG
ACGCACGACTCAATTAACGAGGGTGAAAATGGGCCCACACATCAACCCG
TTGAGTCACCAGCGCTGTTCCGCGCTATGCCAAATATTTACTACATGCGCC
CGGTTGACTCCGCAGAAGTGTTCGGTCTTTTTCAGAAGGCAGTCGAACTT
CCATTCTCCTCCATCCTGTCGCTTTCACGTAATGAGGTATTGCAATATCCA
GGGAAGTCCTCCGCAGAGAAAGCGCAACGCGGTGGGTACATCCTGGAAG
ACGCGGAGAATGCCGAAGTGCAAATCATTGGAGTCGGGGCCGAAATGGA
ATTTGCTTACAAGGCTGCGAAAATCCTTGGTCGCAAGTTTCGCACTCGCG
TTTTATCAATCCCTTGCACTCGTTTATTTGATGAGCAATCGATTGGATACC
GTCGTAGCGTGCTTCGTAAGGATGGCCGCCAGGTACCCACTGTAGTAGTG
GATGGCCACGTCGCATTCGGCTGGGAACGTTACGCAACCGCAAGCTATTG
TATGAATACTTATGGAAAGTCATTACCTCCCGAGGTAATTTATGAATACTT
TGGGTATAATCCGGCGACTATCGCGAAGAAGGTGGAGGCTTACGTACGC

GCTTGCCAGCGCGACCCCTTATTATTACATGATTTCTTAGACTTAAAAGAG
AAGCCAAACCACGACAAAGTGAATAAACTTTAA
